# High expression of octamer transcription factor 1 in cervical cancer

**DOI:** 10.3892/ol.2014.2023

**Published:** 2014-04-02

**Authors:** SONGSHU XIAO, SHAN LIAO, YANHONG ZHOU, BIN JIANG, YUERAN LI, MIN XUE

**Affiliations:** 1 Department of Gynecology and Obstetrics, The Third Xiangya Hospital, Central South University, Changsha, Hunan 410013, P.R. China; 2 Molecular Genetics Laboratory, Cancer Research Institute, Central South University, Changsha, Hunan 410078, P.R. China

**Keywords:** cervical cancer, octamer transcription factor 1, gene expression

## Abstract

Cervical carcinoma is the second most prevalent malignancy in females worldwide. The crucial etiologic factors involved in the development of cervical carcinoma include infection with papillomavirus, and the structural or functional mutation of oncogenes and tumor suppressor genes. The abnormal change of octamer transcription factor 1 (OCT1) is associated with tumor progression and a poor patient survival rate. However, little is known regarding the effect of OCT1 in cervical cancer. In the present study, flow cytometry, western blot analysis and quantitative polymerase chain reaction (qPCR) were peformed to identify differentially expressed OCT1 in cervical cancer tissue and adjacent non-cancerous tissues. The normalized OCT1 gene expression in cervical cancer was 5.98 times higher compared with the adjacent non-cancerous tissues. Western blot analysis and flow cytometry assessed the levels of OCT1 protein. The results of these two differential techniques showed that the protein expression level of OCT1 was greater in cervical cancer tissues, which corresponded with the qPCR results. Finally, as OCT1 is a potential target gene for microRNA (miR)-1467, -1185, -4493 and -3919, their expression levels were analyzed in cervical cancer tissues and adjacent non-cancerous tissues; they were downregulated by ~45% in the cervical cancer samples. The results of the present study showed that OCT1 is highly expressed in cervical cancer tissues and indicated that OCT-1 may be significant in cervical cancer.

## Introduction

Cervical carcinoma is the second most prevalent and the fifth most fatal malignancy observed in females worldwide. Furthermore, invasion and metastasis are the predominant causes of cancer-associated mortality (1). Consistent infection with high-risk variations of human papillomavirus (HPV) may cause cervical cancer, however, for the progression from a pre-cancerous disease to an invasive cancer, genetic and epigenetic modifications are required. DNA methylation is an early and recurrent molecular modification in cervical carcinogenesis. Dysregulated activation of numerous genes, including cluster of differentiation 44 and SOX9, has been indicated in cervical cancer, however, the mechanism of its regulation in human cervical cancer cells remains elusive (2–4). It has been shown that inactivation of tumor suppressor genes and activation of oncogenes is significant in carcinogenesis and is caused by the genetic and epigenetic alterations. MicroRNAs (miRs) are closely associated with the incidence and regulation of cervical cancer (5). A previous study evaluated the correlation between the risk of cancer with miRNA single nucleotide polymorphisms and no correlation was determined (6). Thus, the etiology of cervical carcinoma remains poorly understood.

Octamer transcription factor 1 (OCT1) is a ubiquitous member of the Pit-Oct-Unc-homeodomain family. OCT1 has been indicated in metabolic control, stress responses and transcription states, and it also regulates normal and pathological stem cell function. A study by Maddox *et al* (7) demonstrated that a reduced expression of OCT1 by RNA interference results in a reduction of the proportion of aldehyde dehydrogenase 1 (ALDH) (HI) and dye efflux (HI) cells, whereas an increase in OCT1 increases the proportion of ALDH (HI) cells. OCT1 promotes the tumor engraftment frequency and the potential of hematopoietic stem cell engraftment in competitive and serial transplants (7). An additional study revealed that methylation of the OCT1 gene in human esophageal cancer cells is induced by long-term cisplatin exposure, resulting in cisplatin resistance (8). The abnormal change of OCT1 is associated with tumor progression and a poor patient survival rate (9). However, little is known regarding the effect of OCT1 in cervical cancer.

In the present study, quantitative polymerase chain reaction (qPCR) was performed to identify differentially expressed OCT1 in cervical cancer and adjacent non-cancerous tissues. Western blot analysis and flow cytometry were conducted to assess the expression levels of OCT1 protein. As OCT1 is a potential miR-1467, -1185, -4493 and -3919 target, OCT1 expression levels were analyzed in cervical cancer tissues and adjacent non-cancerous tissues to assess its involvement in cervical cancer.

## Patients and methods

### Tumor samples

In total, 10 participants were recruited for the present study from The Third Xiangya Hospital, Central South University (Changsha, China). Consent forms were obtained from the individual patients and experimental protocols were approved by the Institutional Review Board of The Third Xiangya Hospital. The 10 participants were Chinese females with histologically-confirmed cervical cancer (Table I). Cervical cancer tissues and adjacent non-cancerous tissues were collected and each biopsy sample was divided into two sections; one was submitted for routine histological diagnosis and the remaining section was used for qPCR, western blot and flow cytometric analysis.

### RNA extraction and qPCR analysis

Total RNA was extracted from the biopsy samples using a RNeasy kit (Qiagen, Carlsbad, CA, USA) according to the manufacturer’s instructions. The total RNA samples (1 μg) were used to generate cDNA. The PCR reaction was conducted following the reverse transcription reaction. All qPCR reactions were repeated at least three times with varying numbers of extension cycles to avoid false results. Glyceraldehyde-3-phosphate dehydrogenase (GAPDH) served as an endogenous control for normalization. The sequences of the primers used for qPCR were as follows: Forward, 5′-cctgcctcgtcatgattttt-3′ and reverse, 5′-acgaatgtggggtacagctc-3′ for OCT1; and forward, 5′-cgaccactttgtcaagctca-3′ and reverse, 5′-actgagtgt ggcagggactc-3′ for GAPDH. The expression of mRNA was assessed by evaluating the threshold cycle (CT) values. The CT values were normalized with the expression levels of GAPDH and the relative amount of mRNA specific to each of the target genes was calculated using the 2^−ΔΔCT^ method (10–12).

### Western blot analysis

Protein from the biopsy samples was prepared using lysis buffer. The protein concentrations were determined using the bicinchoninic acid (Pierce Chemical, Rockford, IL, USA) protein assay method. The extracts containing 50 μg protein were separated in 10% SDS-PAGE gels and electroblotted onto nitrocellulose membranes (Hyclone Laboratories, Logan, UT, USA). The membranes were blocked using Tris-buffered saline and Tween 20 (25 mM Tris-HCl, 150 mM NaCl, pH 7.5, and 0.05% Tween 20) containing 5% non-fat milk followed by an overnight incubation at 4°C with primary antibodies (rabbit anti-OCT1 antibody, 1:500; Santa Cruz Biotechnology, Inc., Santa Cruz, CA, USA). Following three washes with PBS, the membranes were incubated with the horseradish peroxidase-conjugated secondary antibodies (1:2,000; Santa Cruz Biotechnology, Inc.) and the specific signals were visualized using an enhanced chemiluminescence detection system (Universal Hood II, Molecular Imager ChemiDoc XRS+, Bio-Rad, Hercules, CA, USA). The anti-GAPDH antibody (1:3000; Santa Cruz Biotechnology, Inc.) served as a loading control.

### Intracellular protein level detection by fluorescence-activated cell sorting (FACS)

Single-cell suspensions of cervical cancer tissues or adjacent non-cancerous tissues were prepared. Enzymatic digestion was incubated at 37°C until full digestion had occurred, with oscillations every 10–15 min prior to passing the sample through a 70-μm cell strainer. The resulting cell suspension was centrifuged (Eppendorf 5417C; Eppendorf, Engelsdorf, Germany) at 500 × g for 10 min and resuspended in saline. The cells were fixed in 500 μl paraformaldehyde 4% in Dulbecco’s phosphate-buffered saline (D-PBS) for 20 min at room temperature. Subsequent to washing in D-PBS, the cells were permeabilized with detergents (Triton X-100). The cells were washed twice with D-PBS, and the single-cell suspensions were stained and incubated at 4°C for 30 min with fluorescein isothiocyanate (FITC)-conjugated OCT-1 (Biorbyt, Cambridge, UK). Isotype controls were performed with an FITC-conjugated rabbit anti-human igG negative control (Biorbyt). All antibodies were used according to manufacturer’s instructions. The cells were washed twice and examined by FACS using a MoFlo™ XDP High-Performance Cell Sorter (Beckman Coulter, Miami, FL, USA). Data were acquired and analyzed using Summit v5.2 software (Becton Dickinson, Franklin Lakes, NJ, USA).

### Expressions analysis of miR-1467, -1185, -4493 and -3919 in cervical cancer

The total RNA was extracted from the biopsy samples with the RNeasy kit according to the manufacturer’s instructions. cDNA was synthesized from 2 mg total RNA with moloney murine leukemia virus (M-MLV) Reverse Transcriptase (Promega Corporation, Madison, WI, USA) in 25 ml [2 mg total RNA, 400 mM reverse transcription primer oligo(dT)18 for random primers for U6 rRNA and miR-1467, -1185, -4493 and -3919 specific primers (Bulge-Loop™ miRNA qPCR Primers; RiboBio, Co., Ltd., Guangzhou, China) for miRNA, 4 U/ml M-MLV, 1 U/ml inhibitor and 0.4 mM dNTP mix]. qPCR was carried out with the reagents of a Sybr green I mix (Takara Bio, Co., Inc., Dalian, China) in a 20-ml reaction volume (10 ml Sybr green I mix, 200 mM forward and reverse primer and 2 ml cDNA template) on an MJ Opticon Monitor Chromo4™ instrument (Bio-Rad, Hercules, CA, USA) using the following protocol: 95°C for 20 sec and 40 cycles of 95°C for 10 sec, 60°C for 20 sec and 70°C for 1 sec. Data analysis were performed using the 2^−ΔΔCT^ method (10–12).

### Statistical Analysis

Differences of non-parametric variables were analyzed by Fisher’s exact test using EPI software (EPI Info, version 3.2.2; www.CDC.gov/epiinfo/). Differences of the quantitative variables between groups were analyzed by Student’s t-test using the SPSS 11.0 program (SPSS, Inc., Chicago, IL, USA) and P<0.05 was considered to indicate a statistically significant difference.

## Results

### Detection of mRNA expression levels of the OCT1 gene in cervical cancer

In the present study, all 10 cervical cancer tissues samples were squamous cell cancer. There was a 90% (9/10) infection rate of HPV 16 or 18. Other HPV types included HPV 6, 53, and 58. In addition, there were 20% peasants (2/10; Table I) (13).

**Figure 1:**
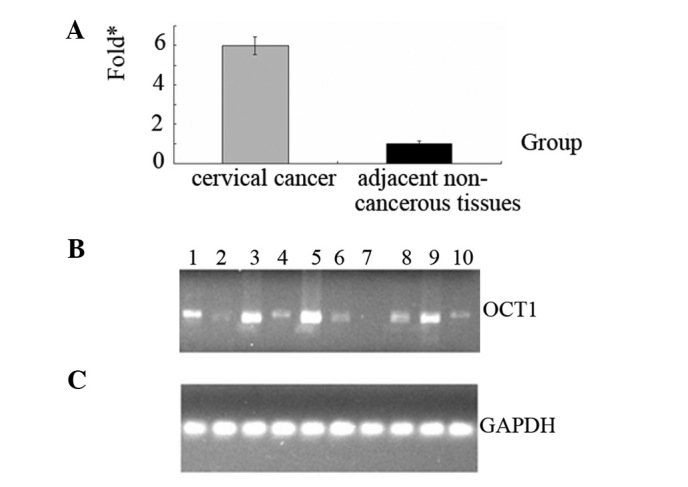
Differential expression of the OCT1 gene in cervical cancer and the adjacent non-cancerous tissues. (A) Normalized OCT1 gene expression in cervical cancer was 5.98 times higher (^*^fold change) compared with the adjacent non-cancerous tissues. (B and C) The results of agarose gel electrophoresis of qPCR for OCT1 and GAPDH genes in cervical cancer (lanes 1, 3, 5, 7 and 9) and the adjacent non-cancerous tissues (lanes 2, 4, 6, 8 and 10). OCT1, octamer transcription factor 1; GAPDH, glyceraldehyde-3-phosphate dehydrogenase; qPCR, quantitative polymerase chain reaction.

To detect the mRNA expression levels of the OCT1 gene in cervical cancer and the adjacent non-cancerous tissues, 10 samples of each were selected to perform qPCR of the OCT1 gene. The data were analyzed using the 2^−ΔΔCT^ method and the fold change in the expression of the OCT1 gene relative to the internal control gene, GAPDH, was analyzed. The expression of the OCT1 gene was higher in the cervical cancer samples compared with the adjacent non-cancerous tissues (Table II, Fig. 1) and the normalized OCT1 gene expression in cervical cancer was upregulated by 5.98 fold (Fig. 1A). The results of agarose gel electrophoresis of qPCR for the OCT1 and GAPDH genes in cervical cancer and the adjacent non-cancerous tissues is shown in Fig. 1B and C.

### Western blot analysis of protein expression levels of the OCT1 gene in cervical cancer

**Figure 2:**
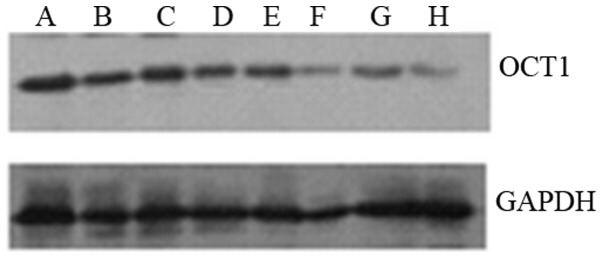
Expression levels of the OCT1 protein in cervical cancer and the adjacent non-cancerous tissues. In total, (lanes A, C, E and G) four cervical cancer and (lanes B, D, F and H) four of the adjacent non-cancerous tissues were selected to detect the expression levels of OCT1 protein by western blot analysis. Data are representative of three independent experiments. OCT1, octamer transcription factor 1; GAPDH, glyceraldehyde-3-phosphate dehydrogenase.

To determine whether the OCT1 gene was expressed at a higher level in cervical cancer compared with the adjacent non-cancerous tissues, the protein expression levels of OCT1 were further examined by western blot (Fig. 2). In comparison with the adjacent non-cancerous tissues, the expression level was identified to be greater in cervical cancer tissues, which corresponded with the qPCR results. These results identified that OCT1 is highly expressed in cervical cancer.

### FACS analysis of protein expression levels of the OCT1 gene in cervical cancer

**Figure 3:**
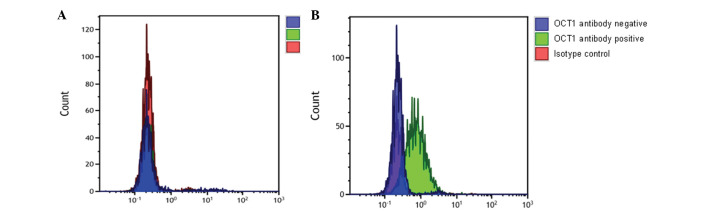
Analysis of the protein expression levels of OCT1 in psoriasis by FACS. The expression levels of the OCT1 protein were tested by FACS in 10 cervical cancer and adjacent non-cancerous tissues. (A) Adjacent non-cancerous and (B) cervical cancer tissues sample. The green, red and purple colors show the results of samples dyed with FITC-conjugated OCT1 antibody, FITC-conjugated rabbit anti-human IgG negative control and FITC-conjugated OCT1 antibody, respectively. Data are representative of three independent experiments. OCT1, octamer transcription factor 1; FACS, fluorescence-activated cell sorting; FITC, fluorescein isothiocyanate.

To further identify that OCT1 is highly expressed in cervical cancer tissues, the protein expression levels of OCT1 in cervical cancer tissues and the adjacent non-cancerous tissues were examined by FACS (Fig. 3). In comparison with the adjacent non-cancerous tissues, the expression level was greater in the cervical cancer tissues. This corresponds with the qPCR results and further identifies that OCT1 is highly expressed in cervical cancer tissue.

### Expression of miR-1467, -1185, -4493 and -3919 is downregulated in cervical cancer

As OCT1 is a potential miR target, the open access programs, TargetScan (http://www.targetscan.org/), PicTar (http://pictar.mdc-berlin.de/) and miRBase (http://mirbase.org/index.shtml), were used to predict the targets of miR-1467, -1185, -4493 and -3919. The endogenous expressions of miR-1467, -1185, -4493 and -3919 were compared between the cervical cancer tissues and adjacent non-cancerous tissues by qPCR. As shown in Table III, the expression of miR-1467, -1185, -4493 and -3919 were downregulated by ~45% in the cervical cancer tissues. These results indicate that OCT1 may be a putative target for cervical cancer.

## Discussion

Cervical cancer is the second most common cause of cancer-associated mortality among females worldwide and in China, subsistence farmers and farm labourers show a higher incidence of cervical cancer than other occupations. The development of novel strategies for diagnosis, prognosis and treatment requires consideration. There have been numerous attempts at designing novel therapeutic agents and developing strategies for immunotherapy and gene therapy for the treatment of cervical cancer (14,15). Specific biomarkers are required for the early diagnosis and prediction of metastatic progression and effective therapy. However, there is currently no efficient therapy against cervical cancer and the available treatments have various disadvantages (1,16–21). It has been shown that inactivation of tumor suppressor genes and activation of oncogenes is significant in carcinogenesis, and results from genetic and epigenetic alterations (5,6). However, the etiology of cervical carcinoma remains poorly understood.

The OCT1 transcription factor was among one of the first identified members of the POU transcription factor family. Members of this family contain the POU domain, a 160-amino acid region necessary for DNA binding to the octameric sequence ATGCAAAT. Oct-1 controls the transcriptional regulation and affects tumor development (9). The results of the present study showed that the expression levels of the OCT1 gene in cervical cancer was 5.98 times higher compared with adjacent non-cancerous tissues. Furthermore, the protein expression level of OCT1 was shown to be higher in cervical cancer by two differential techniques, western blot analysis and flow cytometry. These results correspond with the results of the qPCR. The expression of miR-1467, -1185, -4493 and -3919 were downregulated by ~45% in the cervical cancer tissues. The results showed that OCT1 was highly expressed in cervical cancer tissues and indicates that OCT-1 is significant in cervical cancer.

The significant role of OCT1 in numerous malignancies, except cervical cancer, has been demonstrated by previous studies. Gupta *et al* (22) demonstrated the expression of human OCT1 in lymphoma cells and the increased susceptibility of the cells to irinotecan and paclitaxel. OCT1 is a coregulator of the androgen receptor (AR) and can be a prognostic factor for prostate cancer, which may lead to the development of a novel therapeutic intervention. OCT1 regulates cell growth of LNCaP cells and is a prognostic factor for prostate cancer (23,24). OCT1 is a negative regulator of enhancer activity mediated by dihydrotestosterone in a subset of AR-occupied regions (ARORs). AROR enrichment for the OCT-binding, TTGGCAAATA-like motif, may indicate a mechanism that maintains correct AR activity at specific ARORs by OCT1, while expanding AR activity in other ARORs. Therefore, OCT1 may be involved in the regulation of prostate development and cancer progression (25,26). A study by Shakya *et al* (27) demonstrated that OCT1 is an adjustable, bipotential stabilizer of inducible and repressed transcriptional states.

In conclusion, the present study demonstrated that OCT1 was highly expressed in cervical cancer tissues and may be significant in cervical cancer. OCT1 is likely to provide a theoretical evidence for elucidating the pathogenesis of cervical cancer if the mechanisms of CD44 regulating OCT1 expression are clarified in cervical cancer.

## Figures and Tables

**Table I:** Characteristics of female cervical cancer patients diagnosed with squamous cell cancer.

Sample no.	Age, years	HPV type^a^	Laborers^b^
1	60	16,53,58	No
2	46	16	No
3	49	18	Yes
4	47	16	No
5	49	6	No
6	43	16	No
7	48	16	No
8	40	16	No
9	46	16	No
10	60	16	Yes

a HPV types are defined according to the study by Walboomers *et al.*

b Subsistence farmers and farm labourers are listed as in China they show a higher incidence of cervical cancer than other occupations.

HPV, human papillomavirus.

**Table II:** Identification of the mRNA expression level of the OCT1 gene in cervical cancer and adjacent non-cancerous tissues by qPCR.

		CT, means ± standard deviation				
			
Sample	n	GAPDH	OCT1	Δ	ΔΔ	Fold^a^
Cervical cancer	10	16.56±1.32	27.47±1.51	10.91±0.84	−2.58±0.63	5.98
Non-cancerous tissues	10	16.23±1.25	29.72±1.67	13.49±0.92		

a Mean fold change in expression of the target gene, OCT1, relative to the internal control gene, GAPDH, was calculated using the 2^−ΔΔCT^ equation previously adopted by Livak *et al* (10): ΔΔCT = (CT_Target_ - CT_GAPDH_)_cervical cancer_ - (CT_Target_ - CT_GAPDH_)_control_. At least three replicates of each reaction were performed.

CT, threshold cycle; qPCR, quantitative polymerase chain reaction; OCT1, octamer transcription factor 1; GAPDH, glyceraldehyde-3-phosphate dehydrogenase.

**Table III:** Identification of the expression levels of miR-1467, -1185, -4493, and -3919 in cervical cancer and adjacent non-cancerous tissues.

			CT, mean ± standard deviation				
				
miRNA	Sample	n	U6^a^	miRNA	Δ	ΔΔ	Fold^a^
miR-1467	Cervical cancer	10	17.14±0.92	30.88±1.08	13.74±0.93	0.89±0.11	0.53
	Non-cancerous	10	17.52±0.87	30.37±1.17	12.85±1.08		
miR-1185	Cervical cancer	10	19.36±0.94	31.99±1.29	12.63±1.06	0.77±0.09	0.59
	Non-cancerous	10	19.58±0.99	31.44±1.18	11.86±1.03		
miR-4493	Cervical cancer	10	18.71±0.84	30.72±1.22	12.01±0.85	0.71±0.10	0.61
	Non-cancerous	10	18.79±0.79	30.09±1.25	11.30±1.01		
miR-3919	Cervical cancer	10	17.28±0.80	30.12±1.27	12.84±1.01	0.80±0.14	0.57
	Non-cancerous	10	18.19±0.86	30.23±1.23	12.04±1.11		

a U6 was used as a control.

b Expression fold change of miRNA in cervical cancer compared with adjacent non-cancerous tissues.

CT, threshold cycle; miRNA, microRNA.

## Abbreviations

OCT1: octamer transcription factor 1
GAPDH: glyceraldehyde-3-phosphate dehydrogenase
HPV: papillomavirus
